# Complete mitochondrial genome of Taiwanese loach, *Paramisgurnus dabryanus* ssp. (Cobitinae)

**DOI:** 10.1080/23802359.2018.1450665

**Published:** 2018-03-13

**Authors:** Xia Liang, Daoyu Zhu, Xiaodong Li, Kejun Cai, Haili Zhang, Guosong Zhang

**Affiliations:** a Key Laboratory for Physiology Biochemistry and Application, School of Agriculture and Bioengineering, Heze University, Heze, Shandong, China;; b School of Chemistry and Bioengineering, Hechi University, Yizhou, Guangxi, China;; c Department of Life Science, Huzhou University, Huzhou, Zhejiang, China

**Keywords:** Taiwanese loach, Paramisgurnus, mitogenome, conservation

## Abstract

Taiwanese loach was bred in Formosa and then widely cultivated in China. Because of overfishing and environmental pollution, the number of wild Taiwanese loach has been sharply decreased in these years. Also the taxonomic status of Taiwanese loach is still unclear. In this study, the complete mitochondrial genome of Taiwanese loach was obtained by PCR. The genome is 16,569 bp in length, including 2 ribosomal RNA genes, 13 proteins-coding genes, 22 transfer RNA genes, and a non-coding control region, the gene composition and order of which was similar to most reported from other vertebrates. Sequence analysis showed that the overall base composition is 29.5% for A, 27.5% for T, 26.4% for C, and 16.6% for G. The sequence is a slight A + T bias of 57.0%. By analyzing phylogenetic analysis and BLAST, the similarity to Paramisgurnus was >99%, we dare to speculate that the Taiwanese loach cultivated in China was a subspecies of *P. dabryanus*. Mitogenome information from this study could be a useful basis for conservation and phylogenetics of Taiwanese loach, *P. dabryanus* ssp.

The Taiwanese loach is a commercially important Cypriniformes fish species in Asia. Taiwanese loach was bred in Formosa and then widely cultivated in some provinces of China. As with many other cultured fish species, the seedling production by catching wild parent ways, the multiple generations of inbreeding, and various diseases for Taiwanese loach have given rise to degeneration of genetic characterization, its output presents on a declining curve for years running (Yang et al., 2017). Because of overfishing and environmental pollution, the number of wild Taiwanese loach has been sharply decreased in these years. Also the taxonomic status of Taiwanese loach is still unclear. Therefore, it is very important to characterize the complete mitogenome of this species, which could be a fundamental basis to address genetic identity and diversity in future conservation program of this rarely occurring species (Haddad et al. 2017). In this study, we sequenced the complete mitogenome of Taiwanese loach with a GenBank accession number MG725379. The voucher specimen was collected from Zhili Fanyi aquaculture base, north latitude 30°22″ and east longitude 120°25″, Huzhou city, China, which were stored in biology herbarium of Heze University. Its tailfins were preserved in 95% alcohol. All DNA were extracted using phenol–chloroform extraction methods and stored at −80 °C. The mitogenome were amplified by primers which were initially published *Paramisgurnus dabryanus* (Dai et al. 2016).

The overall base composition was 29.5% for A, 27.5% for T, 26.4% for C, and 16.6% for G. The sequence was a slight A + T bias of 57.0%. Most of the protein genes used ATG as the initiation codons (ND1, ND2, COX2, ATP8, ATP6, COX3, ND3, ND4L, ND4, ND5, Cytb), except for COX1 and ND6 genes, which used GTG and ATA instead of ATG. Eight protein-coding genes ended with complete termination codons, TAA (ND1, ND2, COX1, ATP8, ATP6, ND3, ND4L, and ND5), gene ND4 used TAG as termination codons, and ND6 use CAT instead of TAA. COX2, COX3 and Cytb shares the incomplete stop codons T. Except for eight tRNA (tRNA^Ser^, tRNA^Pro^, tRNA^Glu^, tRNA^Tyr^, tRNA^Cys^, tRNA^Asn^, tRNA^Ala^, tRNA^Gln^) and the ND6 genes encoded on the L-strand, the other genes were encoded on the H-strand. The 21 kinds of tRNA, tRNA^Leu^ and tRNA^Ser^ repeated in the complete mitochondrial genome, respectively. This feature is similar to other fish mitochondrial genes. The complete mitgenome sequence of Taiwanese loach had 16s RNA (1678 bp) and 12s RNA (953 bp), which were located between tRNA^Phe^ with tRNA^Leu(UUR)^ and were separated by tRNA^Val^ genes. The location is same with most vertebrates. As in most vertebrates, two non-coding regions were found in Taiwanese loach mitogenome, the only CR (913 bp) gene located between tRNA^Pro^ and tRNA^Phe^, and an OL (30 bp) was located between tRNA^Asn^ and tRNA^Cys^. The 21 tRNA genes, ranging from 66 to 76 bp in size, except for tRNA^Ser (AGY)^, which lacks a dihydrouridine arm, could be folded into cloverleaf secondary structure. The origin of L-strand was in the WANCY region including five tRNA genes (tRNA^Trp^, tRNA^Ala^, tRNA^Asn^, tRNA^Cys^ and tRNA^Tyr^) and it can fold into a stem-loop secondary structure with the conserved motif 50-GCCGG-30.

To determine taxonomic status of Taiwanese loach, we performed the phylogenetic relationship of this loach stock with other natural populations in loach as inferred by entire mitogenome (Sang et al. 2017). The phylogenetic tree showed that Taiwanese loach to be one of Paramisgurnus, and the other loaches had their own branches (Figure 1). Also the mitochondrial genome sequence of Taiwanese loach were aligned by BLAST, compared with Cobitinae the sequence similarity could reach >95%, and the similarity to Paramisgurnus was >99%. By above results, we dare to speculate that the Taiwanese loach cultivated in China was a subspecies of *P. dabryanus*.

**Figure 1.**
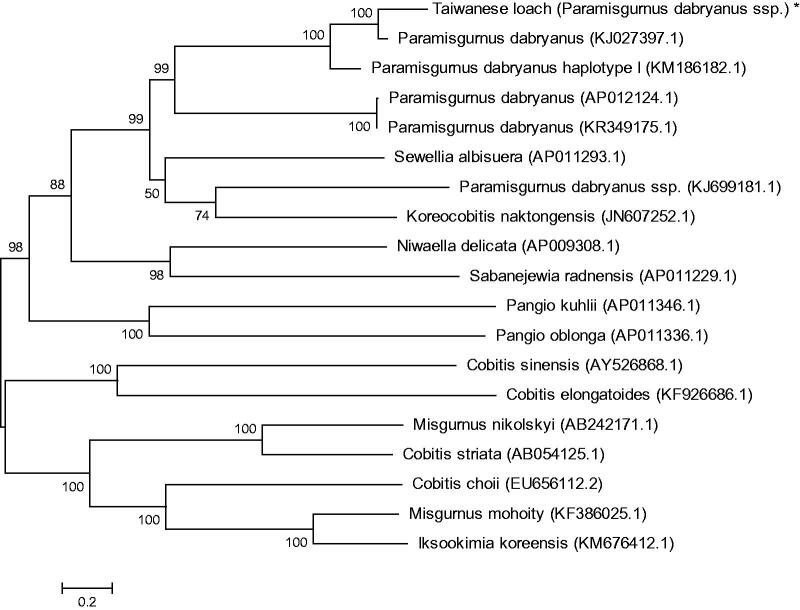
Phylogenetic relationship of Taiwan loach stock with other loach as inferred by entire mitogenome. *The Taiwan loach (accession number: MG725379) in the position of the evolutionary tree. Trees were reconstructed using MEGA 7 program (ver. 7.0.26) with neighbour-joining method. Numbers above branches are bootstrap values by 1000 replicates. The phylogenetic tree showed that Taiwanese loach to be one of Paramisgurnus, and the other loaches had their own branches (e.g. Cobitis, Pangio, Misgurnus).
